# Impact of convection on the damping of an oscillating droplet during viscosity measurement using the ISS-EML facility

**DOI:** 10.1038/s41526-021-00166-4

**Published:** 2021-10-05

**Authors:** Xiao Xiao, Jürgen Brillo, Jonghyun Lee, Robert W. Hyers, Douglas M. Matson

**Affiliations:** 1 Institut für Materialphysik im Weltraum, Deutsches Zentrum für Luft- und Raumfahrt (DLR), Köln, Germany; 2 Department of Mechanical Engineering, Iowa State University, Ames, IA USA; 3 Department of Mechanical and Industrial Engineering, University of Massachusetts Amherst, Amherst, MA USA; 4 Department of Mechanical Engineering, Tufts University, Medford, MA USA

**Keywords:** Fluid dynamics, Materials science, Engineering

## Abstract

Oscillating droplet experiments are conducted using the Electromagnetic Levitation (EML) facility under microgravity conditions. The droplet of molten metal is internally stirred concurrently with the pulse excitation initiating shape oscillations, allowing viscosity measurement of the liquid melts based on the damping rate of the oscillating droplet. We experimentally investigate the impact of convection on the droplet’s damping behavior. The effective viscosity arises and increases as the internal convective flow becomes transitional or turbulent, up to 2–8 times higher than the intrinsic molecular viscosity. The enhanced effective viscosity decays when the stirring has stopped, and an overshoot decay pattern is identified at higher Reynolds numbers, which presents a faster decay rate as the constraint of flow domain size becomes influential. By discriminating the impact of convection on the viscosity results, the intrinsic viscosity can be evaluated with improved measurement accuracy.

## Introduction

The oscillating droplet method allows the study of the thermophysical properties of liquids using an electromagnetic levitation (EML) technique in microgravity^1–6^ onboard the International Space Station (ISS) with the ISS-EML facility^7^. Viscosity is an essential thermophysical property, which is the fundamental property of liquids and a key parameter for fluid flow model development. Thus, accurate measurement of the temperature-dependent viscosity is a requirement for both thermo-fluid studies and industrial applications. EML is a widely used containerless processing technique, which can process the melts of conducting materials such as molten metals, allowing the melts to achieve a wide range of temperature and prevent contamination. During an ideal experiment, a spherical sample is levitated and melted into an undeformed droplet, then deformed by Lorentz forces induced from pulse excitation. The freely damped oscillations of the droplet are used to assess the viscosity of the melts based on Lamb’s theory^8^. Instead of the ground experiments, where the electromagnetically levitated droplet usually forms an asymmetric shape due to gravity, the microgravity conditions allow the droplet to present a near-spherical shape and provide a clearer oscillation signal for better damping analysis. However, the strong internal convective flow will arise when the Lorentz forces simultaneously deform the droplet surface and stir the inner fluids. Previous experimental and numerical studies^1,2,9–12^ showed that convection significantly influences viscosity measurement. The effective viscosity appears anomalously high and can be an order of magnitude higher than the molecular viscosity^10,12^ when eddies are observed in the stirred droplet^13^. Hyers et al.^11^ suggested that the convection could provide an alternate mechanism for the damping of surface oscillation, as the damping time of the oscillating droplet is dominated by eddy dissipation. There still lacks systematic and quantitative investigations on how the effective viscosity changes as the convection becomes influential. It is thus essential to investigate and quantify the onset and decay of effective viscosity of an internally stirred droplet for more accurate measurement of thermophysical properties, providing benchmarks for the development of fluid flow models, and presenting an improvement in the interpretation of physical mechanism regarding liquid droplet subjected to internal flows. In this work, we experimentally determined the magnitude and decay behavior of the effective viscosity in an oscillating droplet under various initial conditions. Two decay patterns were identified, including an overshoot decay and a free decay associated with different ranges of Taylor-Reynolds number. The decay rates and timescales were evaluated based on the droplet’s surface oscillation damping behavior. Finally, the laminar viscosities of selected molten alloys were measured, including a nickel-based superalloy LEK-94 (Ni63.7-Cr6.8-Co7.4-Mo1.4-W1.4-Ta0.9-Re0.9-Al15.2-Ti1.2-Hf0.08(wt.%)^14^) and an industrial alloy stainless steel Fe60-Cr21-Ni19(at.%).

## Results

**Fig. 1:**
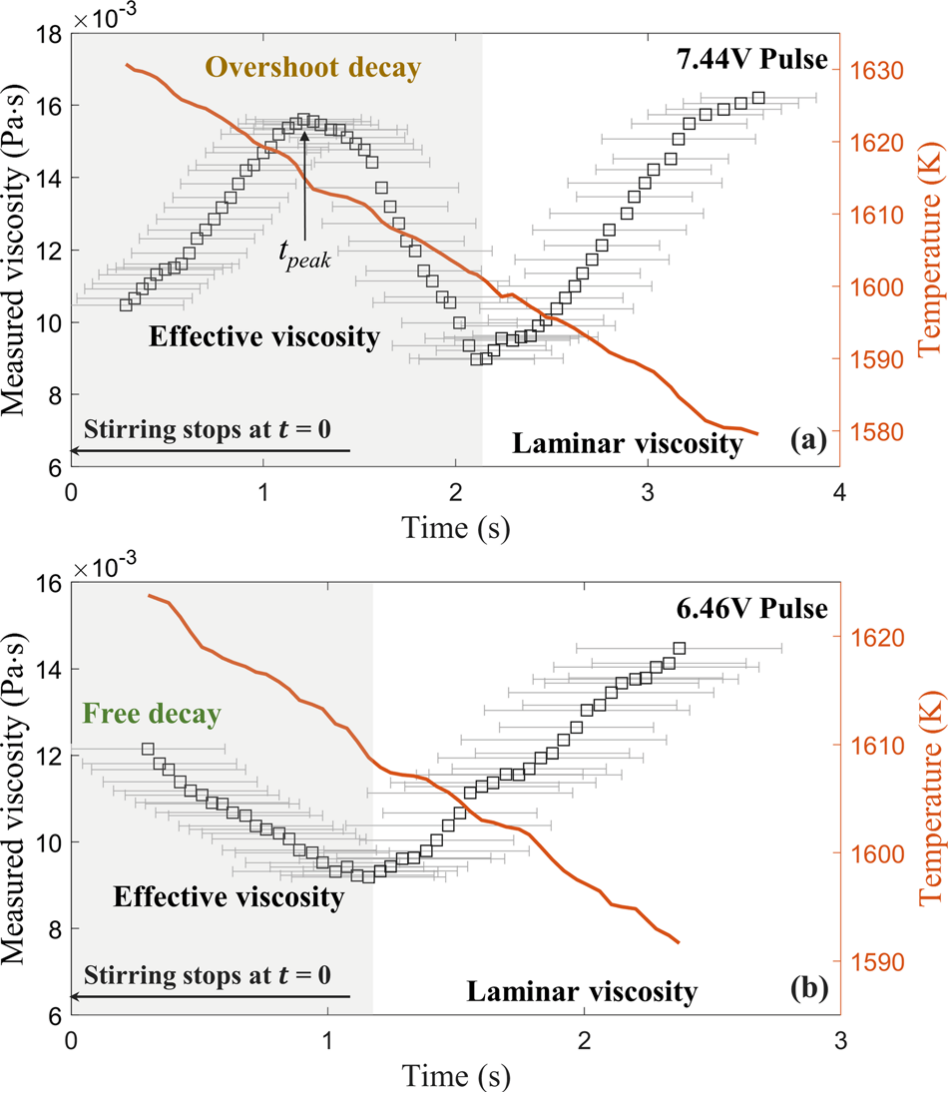
Measured viscosities using the ISS-EML. The examples show the measured viscosities of a molten LEK-94 droplet as a function of elapsed time, superimposed with the decrease in temperature. **a** 7.44 V pulse. **b** 6.46 V pulse.

During the experiments, each excitation pulse will generate Lorentz forces that initiate the oscillation of the droplet and induce the internal convective flow simultaneously. As described in the methods section, the viscosity was measured based on the damped oscillations of the droplet. During the damping process, the internal convective flow will decay for a certain period *t*_eff_ and reduce to a minimum status maintained by the positioning force field. Therefore, the damping consisted of two stages: a convection-dominated stage where the enhanced effective viscosities *μ*_eff_ were measured and a laminar stage where the intrinsic molecular viscosities *μ* were measured. In the first stage, when the damping was dominated by the internal convective flow, the effective viscosities presented significantly higher values than expected; in the second stage, when the flow became very weak, the laminar viscosities increased as the temperature decreased. This two-stage pattern was repeatedly exhibited for every single experimental cycle, as illustrated in Fig. 1 for an example of a molten LEK-94 droplet.

**Fig. 2:**
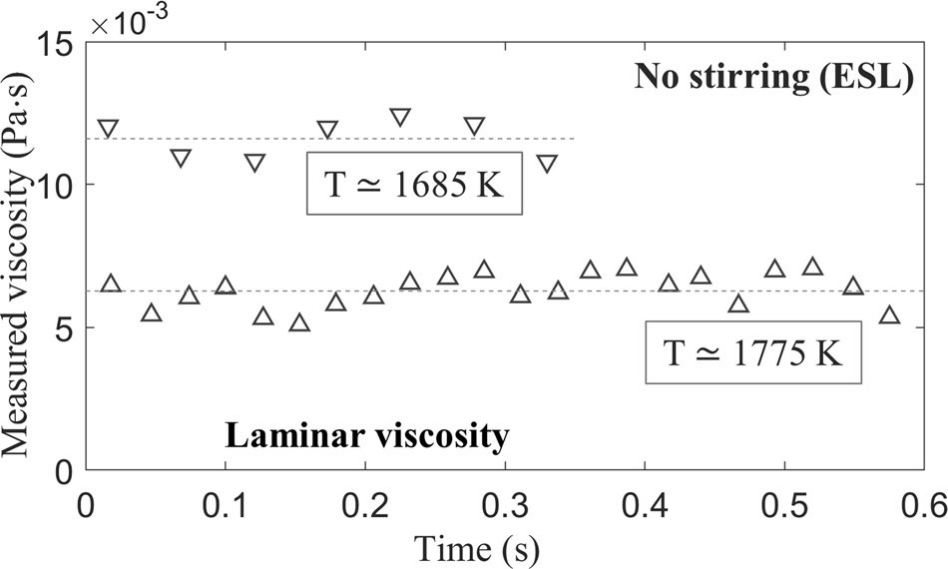
Measured viscosities using the ESL. The example shows the measured viscosities of a molten FeCrNi droplet as a function of elapsed time at constant temperatures.

As a comparison to the ISS-EML tests, the results of electrostatic levitation (ESL) experiments are presented. In ESL, the excitation forces are applied on the droplet surface, and the Reynolds number of the internal flow is less than 110^15^. Figure 2 showed an example of a molten FeCrNi droplet processed in the ESL facility in the NASA Marshall Space Flight Center (MSFC). The molten droplet was electrostatically levitated and heated by a laser, where there is no stirring inside the droplet. The viscosities were measured at different constant temperatures, using the same segmenting method for damping data analysis. The results showed that the measured viscosities fluctuated in a narrow range around specific constant values directly from *t* = 0, indicating that only a laminar stage was exhibited without the existence of stirring.

### Onset of effective viscosity

The effective viscosity changes as a function of time and two decay patterns were observed, for either an overshoot decay or a free decay. As also shown in Fig. 1 for illustration, over a similar temperature range, the example shown in Fig. 1(a) resulted from a 7.44 V pulse represents overshoot decay, where the effective viscosity *μ*_eff_ initially increased and developed to a peak value at time *t*_*peak*_ before decreasing; the example shown in Fig. 1(b) subject to a 6.46 V pulse exhibits free decay, where *μ*_eff_ continuously decayed as the stirring stopped. To characterize the initial conditions as the convection differs from various experimental conditions, the Taylor-Reynolds number ${R}_{\lambda }=\sqrt{20\rho {K}^{2}/3\mu \varepsilon }$ is calculated at *t* = 0, which is averaged over the convection loops. The values of viscosity *μ* and density *ρ* are from the ISS-EML results and the references^16,17^. The kinetic energy *K* and dissipation rate *ε* are obtained from the magnetohydrodynamic (MHD) simulation using a quasi-steady RNG (Re-Normalization Group) *k*-*ε* model for an ISS-EML processed droplet^18^.

**Fig. 3:**
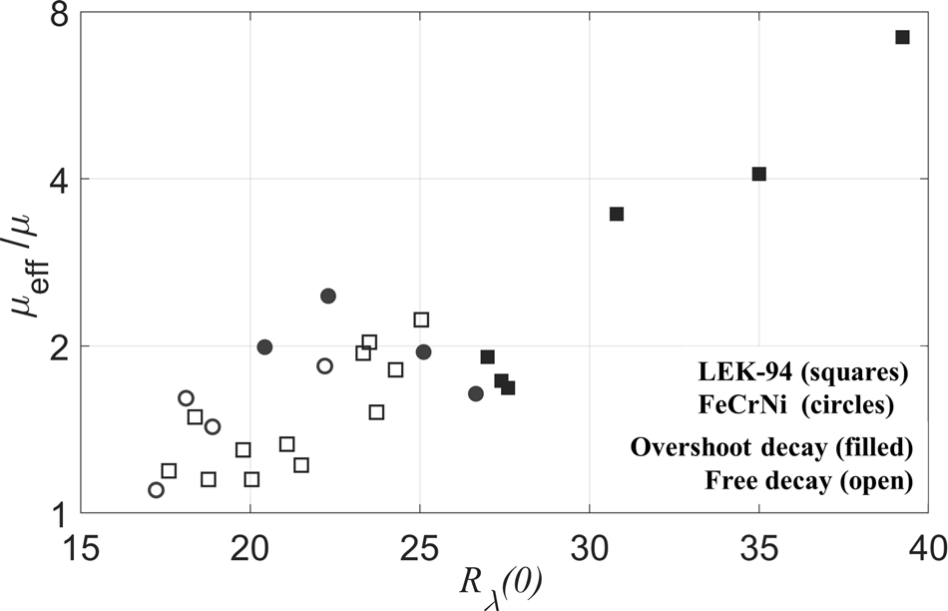
Effective viscosity ratio *μ*_eff_/*μ* (log scale) at t_0_ as a function of R_λ_(0).

The magnitude of effective viscosity ratio *μ*_eff_/*μ* at *t*_0_ is plotted versus different initial conditions *R*_*λ*_(0), as shown in Fig. 3. The ratio *μ*_eff_/*μ* is a metric representing how large the effective viscosity is compared to the intrinsic molecular viscosity, and *μ*_eff_/*μ* at *t*_0_ represents its magnitude when the stirring just stops, where *t*_0_ = 0 for the free decay pattern, and *t*_0_ = *t*_*peak*_ as a virtual origin for the overshoot decay pattern. The effective viscosities *μ*_eff_ exhibit values of 20–60% higher than molecular viscosities with *R*_*λ*_(0) <20, and the effective viscosity ratios *μ*_eff_/*μ* continue to increase to 2–8 times with larger *R*_*λ*_(0).

### Decay of effective viscosity

**Fig. 4:**
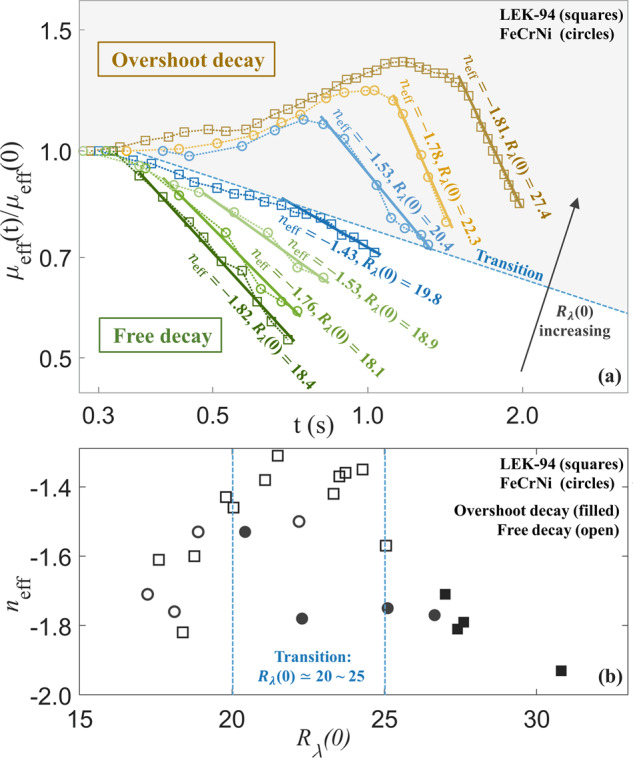
The decaying effective viscosities and effective decay rates for the overshoot and free decay patterns. **a** The representative curves of *μ*_eff_(*t*)/*μ*_eff_(0) on a log-log scale at different *R*_*λ*_(0) along with data fit (solid lines). **b** Effective decay rate *n*_eff_ as a function of *R*_*λ*_(0).

To characterize the decay patterns and determine the decay rate of the effective viscosity, the effective viscosity is normalized to *μ*_eff_(*t*)/*μ*_eff_(0), where *μ*_eff_(0) is the first data point available near the origin of time. Because the underlying molecular viscosities also changed as a function of temperature that is a function of time, *μ*_eff_(*t*)/*μ*_eff_(0) can be corrected by a factor of *μ*(0)/*μ*(*t*). Firstly, as shown in Fig. 4(a), the representative curves of normalized effective viscosities *μ*_eff_(*t*)/*μ*_eff_(0) are plotted versus time *t* at different *R*_*λ*_(0), the following equation is used to determine the effective decay rate *n*_eff_,

1 $${\mu }_{{{{\rm{eff}}}}}(t)/{\mu }_{{{{\rm{eff}}}}}(0)\propto {(t-{t}_{0})}^{{n}_{{{{\rm{eff}}}}}+1},$$

which is a semi-empirical relation that measures how fast the effective viscosity decays. The values of *n*_eff_ are determined from data fit on the log-log scale through a linear least-squares approach. In Fig. 4(a), the shaded area represents the overshoot decay region, and the unshaded area represents the free decay region. It should be noticed that although *n*_eff_ may have similar values in both regions, the time origin *t*_0_ used in Eq. (1) is different. The overshoot decay uses *t*_0_ = *t*_*peak*_, and the free decay uses *t*_0_ = 0, whereas the initial condition *R*_*λ*_(0) is calculated at *t* = 0 for both decay patterns.

As also shown in Fig. 4(b), a free decay pattern will transit to an overshoot decay pattern as *R*_*λ*_(0) increases, and the transition occurs in a critical range of *R*_*λ*_(0) ≃ 20–25. For both decay patterns, the effective decay rates are as fast as *n*_eff_ = −1.82 to 1.93; when *R*_*λ*_(0) moves towards the transition region from either end, the effective decay rates of both patterns are slowing down to *n*_eff_ = −1.31 to −1.46. As an example showing the case near the transition at *R*_*λ*_(0) = 19.8 where *n*_eff_ = −1.43 (Fig. 4(a)), a slight slope change can be observed near *t* = 0.7 s for a total decay time around 1.0 s.

**Fig. 5:**
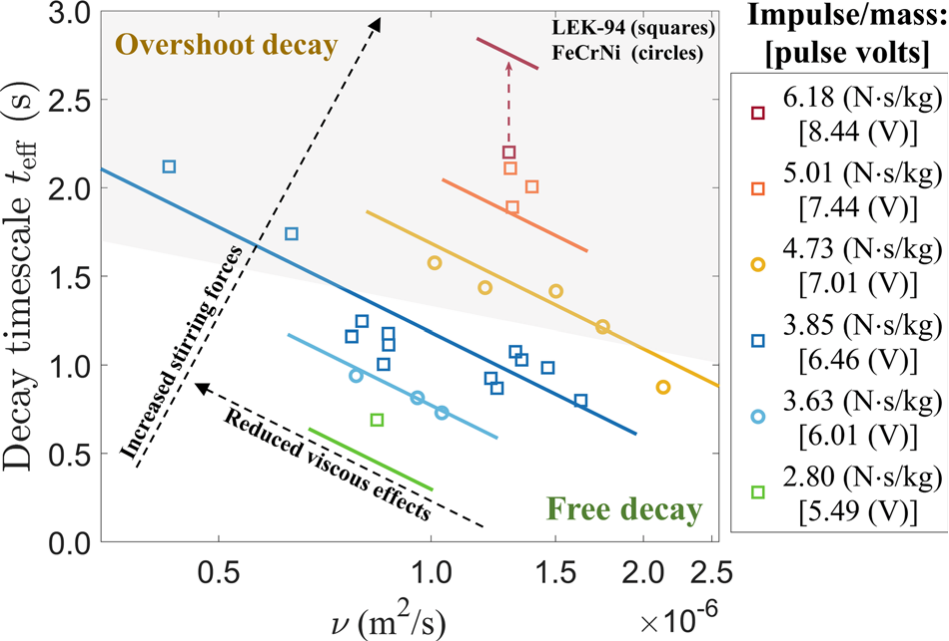
The decay timescale of effective viscosity *t*_eff_ for different experimental cases as a function of kinematic viscosity *ν* (log-scale) and impulse amplitude $\bar{I}$. The solid lines represent the empirical relation obtained from the data fit.

For the decay timescale of effective viscosity *t*_eff_, an empirical relation is developed as a function of the kinematic viscosity *ν* = *μ*/*ρ* at *t* = 0 and the impulse per unit mass $\bar{I}=| {F}_{\delta }| {{\Delta }}t/m$ that transfers into the system,

2 $${t}_{{{{\rm{eff}}}}}\simeq -16.55-0.8568{{\mathrm{ln}}}\,\nu +0.9127{U}_{{{{\rm{ctr}}}}}^{H},$$

where ${U}_{{{{\rm{ctr}}}}}^{H}$ is the pulse voltage and $\bar{I}\simeq 1.0965{U}_{{{{\rm{ctr}}}}}^{H}-3.1249$ (${U}_{{{{\rm{ctr}}}}}^{H} > 5$V). The impulse duration Δ*t* = 0.1 s, and the stirring forces ∣*F*_*δ*_∣ are the pulse voltage correlated Lorentz forces within the skin depth *δ* ≃ 0.4–0.5*R*_0_^19^ of the conductive droplet. It should be noticed that Eq. (2) is an empirical formula, where the coefficients are obtained by data fitting and treated as some physical constants. As shown in Fig. 5, the decay timescales *t*_eff_ at different conditions are displayed along with the fitting lines, which are linear to ${U}_{{{{\rm{ctr}}}}}^{H}$, $\bar{I}$ and the logscale of *ν*; the cases subjected to overshoot decay and free decay are denoted by a shaded and an unshaded region respectively. Longer decay times are expected for either larger impulse amplitude or lower kinematic viscosity that will cause stronger convection with a higher Reynolds number as the initial condition. For an extreme case with impulse $\bar{I}=$ 6.18 N s/kg (${U}_{{{{\rm{ctr}}}}}^{H}=$ 8.44 V, *R*_*λ*_(0) ≃ 35), the droplet oscillation damped out even before it could reach *t*_*peak*_ with a significantly increasing effective viscosity.

### Results of laminar viscosity

**Fig. 6:**
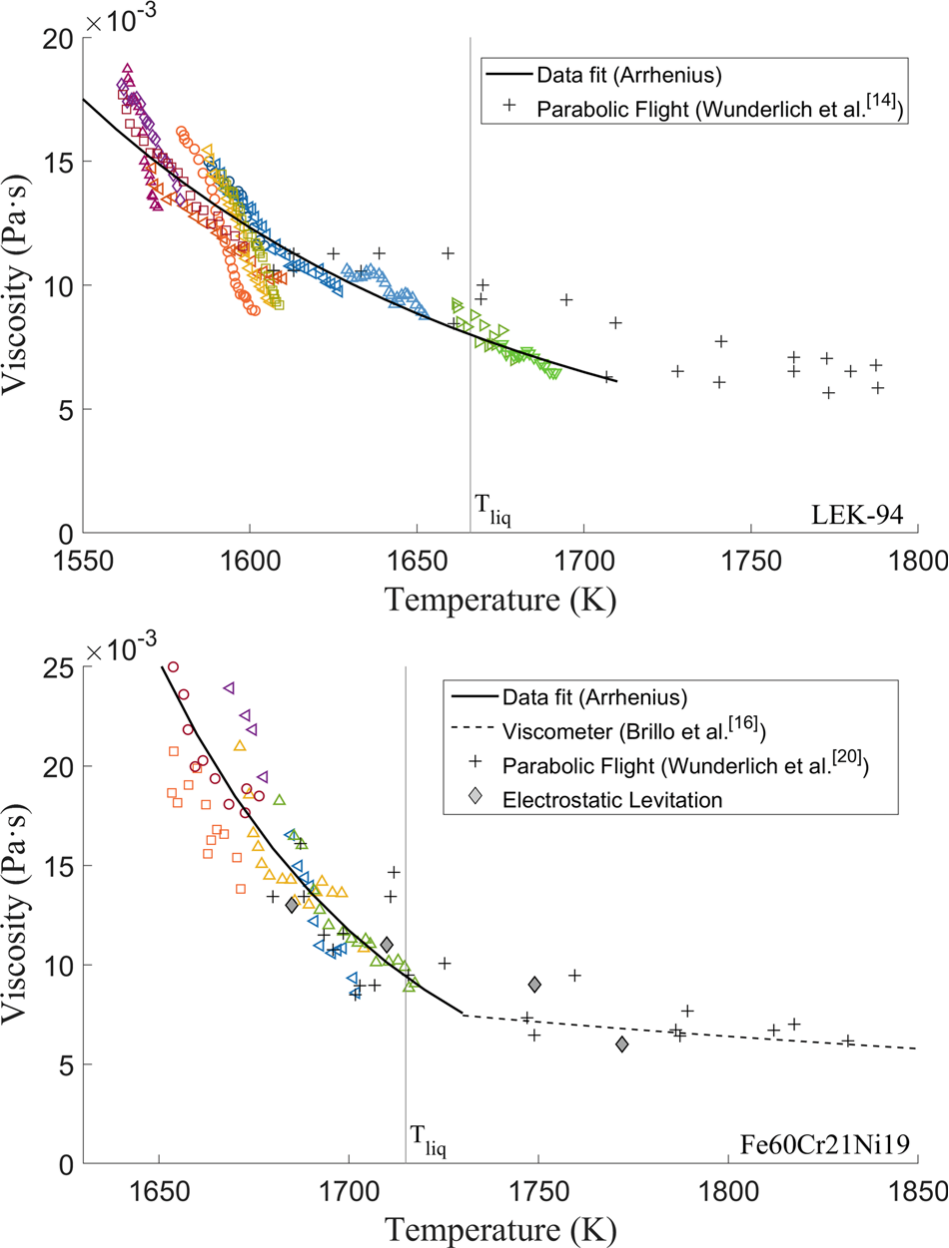
Viscosity as a function of temperature for molten LEK-94 and Fe60Cr21Ni19. Each open symbol represents results from different tests using the ISS-EML; the solid lines represent Arrhenius fit.

The viscosities of molten LEK-94 and Fe60-Cr21-Ni19(at.%) were measured in the laminar stage. The results are presented in Fig. 6 with an Arrhenius fit $\mu =\exp ({p}_{1}/{{{\rm{T}}}}-{p}_{2})$, and the fitting coefficients are displayed in Table 1. The significant digits in the coefficients *p*_1_ and *p*_2_ indicate the standard errors of the least-squares fit between ${{\mathrm{log}}}\,(\mu )$ and 1/*T*. Compared to traditional experimental methods such as the oscillating cup viscometer^16^, more viscosity values in the undercooling region were obtained below the liquidus temperature T_*liq*_; compared to the electrostatic levitation experiments, the viscosities measured in the laminar stage presented an agreement with ESL results under no stirring; compared to the previous results from the reduced-gravity parabolic flight experiments^14,20^, which used similar experimental setups, this work discriminated different stages during the damping process presenting the overestimated effective viscosity and the laminar viscosity, providing an improvement of the viscosity measurement.

**Table 1:** Viscosities of molten alloys (Pa ⋅ s) - Arrhenius fit $\mu =\exp ({p}_{1}/{{{\rm{T}}}}-{p}_{2})$.

Composition	T_*liq*_(K)	*p*_1_	*p*_2_	Range of T(K)
LEK-94	1666	(1.743 ± 0.068) × 10^4^	15.30 ± 0.4	1560–1700
Fe60Cr21Ni19	1715	(4.303 ± 0.487) × 10^4^	29.76 ± 2.9	1650–1720

## Discussion

**Fig. 7:**
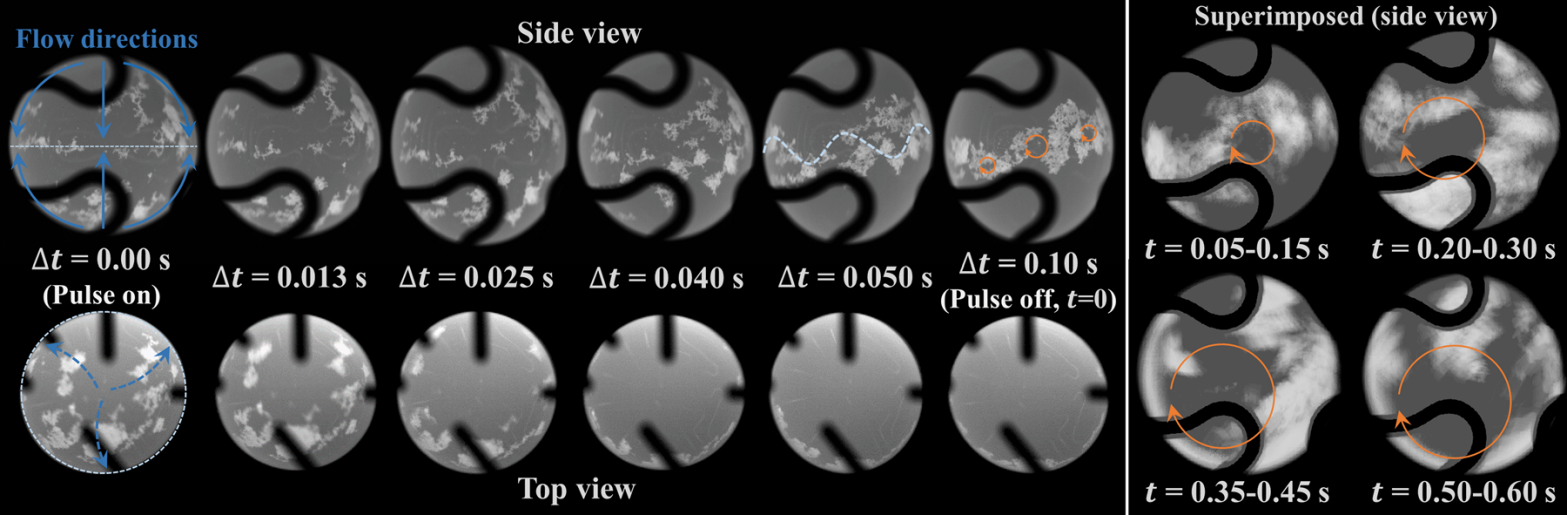
The images of an electromagnetically levitated droplet (CMSX-10). The flow pattern and velocity were traced by the impurity particles on the droplet surface during and after the pulse excitation.

**Fig. 8:**
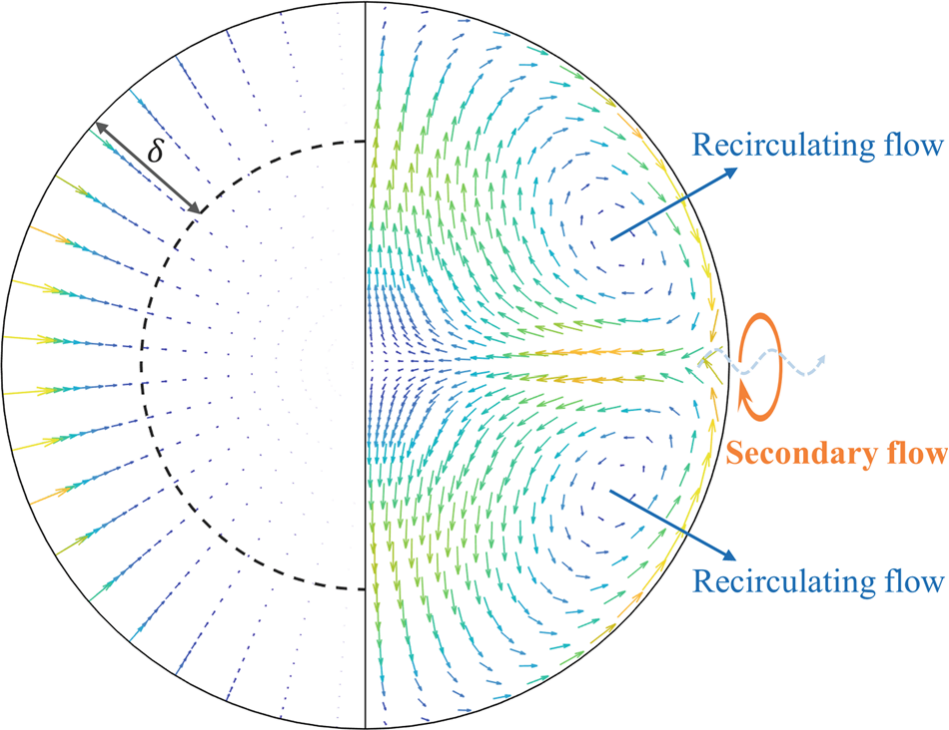
MHD simulation example. (Left side) Distribution of the Lorentz forces. (Right side) Convective flow field.

The pulse excitation-induced internal convective flow plays a leading role in the onset of effective viscosity. To demonstrate the flow pattern of an ISS-EML processed droplet, the particle tracing method was used in another nickel-based superalloy CMSX-10 sample (Ni69.4-Cr2.1-Co3.1-Mo0.5-W5.2-Ta8.3-Re4.7-Al6.3-Ti0.2-Hf0.15-Nb0.1(wt.%)^14^) containing a few surface impurities. A 5.49 V pulse was applied for Δ*t* = 0.1 s at the temperature of 1780 K (*μ* ≃ 6.2 × 10^−3^ Pa ⋅ s, *ρ* ≃ 8.1 × 10^3^ kg/m^3^^14^). The impurity particles were used as tracers to measure the flow velocity along the droplet surface, as shown in Fig. 7 from the side-view and the top-view respectively. The observed flow pattern showed agreement with previous experiment observation^13^ as well as the MHD simulation (Fig. 8). In Figure 8, the left side shows the distribution of the Lorentz forces as the source of stirring, and *δ* denotes the skin depth where the forces are concentrated within this region; the right side shows the convective flow field inside the droplet. A pair of counter-rotating toroidal structures develop symmetrically to the droplet’s equatorial stagnation line; along the droplet surface, the flow moves from either pole towards the equator, where the convective velocity *u* increases to the maximum. This surface velocity directed towards the droplet’s equator is used to calculate the Reynolds number *Re* = *ρuD*/*μ* for a droplet with diameter *D*.

As shown in Fig. 7, during Δ*t* = 0–0.025 s: the tracers started to flow from both semi-spheres directed towards the equator, and the droplet was deformed into a prolate spheroid with the maximum deformation amplitude. During Δ*t* = 0.025 to 0.05 s: the tracers continued to flow towards and gather at the equator while the flow was accelerating. The maximum velocity at Δ*t* = 0.02, 0.03, 0.04, and 0.05 s was about 0.05 m/s, 0.08 m/s, 0.12 m/s, and 0.14 m/s respectively. During Δ*t* = 0.05 to 0.10 s: the tracers showed oscillatory and swirling motion colliding with each other near the equator. Therefore, further tracking of the flow velocity was not possible after Δ*t* >0.05 s, however, the fluid flow was expected to continue developing to approach a quasi-steady status, and the flow velocity at 0.08–0.10 s could be extrapolated to be 0.19–0.20 m/s by a parabolic fit, correlated to *Re* ≃ 830–880, which matches well with the numerical prediction of 0.21 m/s (*Re* ≃ 920) from the MHD prediction^21^. After the pulse was released, the continuous side view images were superimposed by each 0.1 s interval, which showed the development of the structure of the large swirl on the droplet surface. When the stirring stopped at Δ*t* = 0.1 s (*t* = 0), and the flows started to decay at *t* > 0, the large eddy size was increased over time and limited by the boundary of the droplet.

Thus, at Δ*t* = 0.05 s, *u* was about 0.14 m/s (*Re* ≃ 610), the equatorial stagnation line began to become wavy and small eddies can be observed along the equator of the droplet surface as the secondary flow was developing. Shatrov et al.^22^ carried out a stability analysis of the flow inside an electromagnetically levitated droplet and presented a critical Reynolds number of 100–200 where the axisymmetric flow becomes unstable. Ai^23^ studied the flow instability, transition, and turbulence inside an electromagnetically levitated droplet by a three-dimensional direct numerical simulation (DNS). The DNS results^23^ provide more details on the structure of the secondary flows on the droplet surface which are between and perpendicular to the two convective flow loops; the surface flow starts to be unstable at *Re* = 95, and the secondary flow develops at *Re* = 521 as the dividing streamline starts to have a certain up and down pattern that oscillates along the equator, forming a Taylor-Gortler vortex which is a typical flow instability driven by centrifugal forces. The present observation (at *Re* = 610) and the DNS prediction (at *Re* = 521) showed agreement in the confirmation of this flow instability, which was also experimentally observed (at *Re* >500) in previous work by Hyers et al.^13^. Although the instantaneous flow shows some arbitrary characteristics, this particular type of instability can be discriminated from a random chaotic flow. As the Reynolds number continues to increase, the Taylor-Gortler instability leads to a transition to the turbulent flow. Hyers et al.^13^ experimentally characterized the onset of laminar-turbulent transition inside an electromagnetically levitated droplet. The secondary oscillating flow became oscillatory at *Re* >500 and breaking up into observed swirling eddies at *Re* >600, which presents an abrupt and fast hydrodynamic transition of a shear-layer type. This critical Reynolds number is lower than some widely studied flow types such as the Poiseuille flow, Couette flow, or Taylor-Couette flow because the internal flow is constrained by the boundary of the droplet surface in a small bounded domain, intensifying the inertial effect compared to the viscous friction.

In terms of the energy spectrum of turbulence in an electromagnetically levitated droplet, it is hard to be experimentally measured given the available data. However, some characteristics could be inferred by comparing the visible eddy sizes and the theoretical length scales. At *t* = 0s, only small eddies are visible, and the eddy scales are about 0.02–0.04*R*_0_; the Kolmogorov and Taylor length scales are calculated as ${L}_{\eta }={({\nu }^{3}/\varepsilon )}^{1/4}\simeq 0.008{R}_{0}$ and ${L}_{\lambda }=\sqrt{10\nu K/\varepsilon }\simeq 0.076{R}_{0}$. Thus, the small eddy scales are in the same order of magnitude and below the Taylor length scale, indicating the eddy scales appear in the middle between *L*_*η*_ and *L*_*λ*_ that define the inertial range. It is worth noting that this example used the minimum pulse voltage of 5.49 V. If a higher pulse voltage is used, such as the maximum of 8.44 V, the smallest visible eddy sizes might reach the Kolmogorov length scale. It is also possible that the finest eddy structure could only be observable when the tracer particles are very tiny. When the small eddy scales appear through the inertial range from the Taylor length-scale down to the Kolmogorov length-scale where it is responsible for the viscous energy dissipation, it is indicated that the energy cascade process has been reached, which is necessary for the development of turbulence. At *t* > 0.05 s immediately after the stirring has stopped, the larger eddy became visible showing a size greater than 0.23*R*_0_; the integral length-scale is calculated as *L*_*t*_ = *K*^3/2^/*ε* ≃ 0.20*R*_0_, which is comparable to the large eddy size. Thus, the observed eddy scales went through a wide range of length scales over the energy spectrum for a turbulent flow.

For the flow condition inside the droplet, it is difficult to be observed in experiments and will be predicted mainly by the MHD simulations. Different from the overall effective viscosity *μ*_eff_, the distribution of eddy viscosity *μ*_*t*_ over the flow field is not a measurable quantity. The DNS results^23^ showed that the inner symmetric structure of the recirculating loops becomes skewed from *Re* = 521; along the droplet surface, *μ*_*t*_ reaches a local maximum near the equatorial line. Pericleous et al.^24^ developed the spectral collocation method for a dynamic model of an EML oscillating droplet, and Etay et al.^25^ performed the simulation using this model for an oscillating droplet with *R*_0_ = 5 mm and *ν* = 10^−7 ^m^2^/s. The numerical predictions showed that damping of the surface oscillation is much quicker when turbulence exists than in the laminar scenario. The distribution of the values of effective viscosity *μ*_*t*_ inside the droplet indicated that *μ*_*t*_ near the free surface could be larger than the center by an order of magnitude, and the overall effective viscosity ratio *μ*_eff_/*μ* can be over 5. As a result, the turbulence expedites the overall damping rate of the oscillating droplet. Thus, the previous numerical predictions also support the experimental observations showing the impact of convection on the increasing effective viscosity.

The effective viscosity arises as the internal convection becomes transitional or turbulent when the stirring exists and introduces excessive energy, and the effective viscosity decays as the energy starts to decay when the stirring has stopped. The energy decay rate is fundamentally important for the understanding of the dissipation mechanism. From the classical theories, for the free decay of an ideal homogeneous and isotropic turbulence, the kinetic energy *K* usually decays following the power-law^26–29^ with decay rate *n*,

3 $$K(t)\propto {t}^{n}\,.$$

Based on the power-law assumption, the dissipation rate follows

4 $$\varepsilon (t)=-{{{\rm{d}}}}K/{{{\rm{d}}}}t\propto {t}^{n-1}\,.$$

The eddy viscosity *μ*_*t*_ ∝ *ρK*^2^/*ε*, then *ε*(*t*) ∝ *μ*_*t*_(*t*)^(*n*−1)/(*n*+1)^, thus the eddy viscosity *μ*_*t*_ follows the power-law with the exponent *n* + 1,

5 $${\mu }_{t}(t)\propto {t}^{n+1}\,.$$

Theoretically, *n* = −1 for self-similar solutions^26,27^, *n* = −1.2 for Saffman’s −6/5th decay law^28^, *n* = −1.43 for Kolmogorov’s −10/7th faster decay law^29^, and Vassilicos^30^ concluded that more values are theoretically possible depending on different types of invariants. For turbulence decay inside a bounded domain with limited size, Perot^31^ numerically studied the decay rate of mechanically stirred turbulence and inferred that a faster decay rate is expected and will move towards the theoretical limit *n* = − 2 near the boundary^32,33^, as the integral length-scale *L*_*t*_ ∝ *K*^(*n*+2)/2*n*^ increases on the log-log scale over time and eventually converges to a constant value which is limited by the domain size. However, most flows are not ideally homogeneous or isotropic either in nature or engineering applications. There are a few studies on the decay of inhomogeneous or anisotropic turbulence^34–36^. Chasnov^34^ indicated that a more rapid decay is expected with an increasing number of inhomogeneous directions because the energy transports along with those directions. Blay et al.^36^ fitted the power-law to the decay of anisotropic turbulence and experimentally found that the decay rate changes as the turbulence saturation is dominated by the facility size, where *n* = −1.41 for the unsaturated regime (far from the boundary) and *n* = −1.8 for the saturated regime (near the boundary).

For an electromagnetically levitated droplet, the internal flow is homogeneous in the azimuthal direction and inhomogeneous in the radial and polar directions; a two-dimensional anisotropic flow is on the free surface of the droplet. Although there may not be a straightforward relation between the exponent *n* in decay law *μ*_*t*_(*t*) ∝ *t*^*n*+1^ and the effective decay rate *n*_eff_ defined in Eq. (1), which reflects the overall damping behavior of the surface oscillation, there are still a few qualitative characteristics in common. For the free decay pattern of the effective viscosity, the effective decay rate slows down from *n*_eff_ ≃ −1.8 to *n*_eff_ ≃ −1.3 to −1.4 with increasing *R*_*λ*_(0). This observation agrees with a series of numerical results^26,37,38^, showing that a slower decay is expected as the Reynolds number increases. Specifically, characterized by the normalized effective viscosities *μ*_eff_(*t*)/*μ*_eff_(0) over time, the overshoot effect arises with increasing *R*_*λ*_(0), where the transition from free decay pattern to overshoot decay pattern occurs at *R*_*λ*_(0) ≃ 20–25. Usually, when the stirring forces exist, the energy will go through cascades - the inertial transfer of kinetic energy passing through eddies that continuously break into smaller ones until dominated by viscous dissipation; when the stirring has stopped, the energy starts to decay freely.

Oppositely, for the overshoot decay pattern where higher Reynolds number and much stronger convection are exhibited, the excessive incoming energy in a small confined domain will continue to pass through the cascade for a settling time *t*_*peak*_ after the stirring forces have stopped. As a result, a steep drop of the extra kinetic energy and a significant rise of the dissipation rate would be expected before it decreases^27^. Thus, the cascade continued to proceed as a pseudo-forced stirring case; after *t*_*peak*_ it acted to be a free decay. In comparison with this observation, Yoffe et al.^39^ numerically predicted similar decay patterns characterized by the normalized dissipation rate *ε*(*t*)/*ε*(0) from grid-generated turbulence and presented a critical *R*_*λ*_(0) located in the middle between 12.9 and 25.8, indicating that the overshot pattern appears for *R*_*λ*_(0)>25. It may still need further development of the mechanism to explain the decreasing trend of the decay speed towards the transition region. There could be interactive effects between the active and passive loop^13^ of the toroidal flow structures inside the droplet. The recirculating flow near the boundary inside the skin depth is very active and forms the active loop, the viscous interactions with the inner fluid form the passive loop. Different *R*_*λ*_(0) numbers may lead to slightly different interactions between the flows at the droplet surface layer (active loop, near the boundary) and inside the droplet (passive loop, far from the boundary). When *R*_*λ*_(0) reaches the critical range between 20 and 25, the non-cascading dissipation near the boundary may become influential and slow down the overall decay rate. At much higher *R*_*λ*_(0)>25, the active flows are dominated and saturated the near-boundary region, where the growth of large eddies is constrained by the domain size and a faster decay is presented.

In conclusion, the convective flow inside the droplet significantly influences the damping of its surface oscillation. The enhanced effective viscosity increases with the Reynolds number and can be 2–8 times higher than the laminar viscosity. For an electromagnetically levitated droplet, the Lorentz forces internally stir the droplet and form two recirculating convection loops, which induce secondary flows along the equatorial stagnation line, and the instability leads to the laminar-turbulent flow transition. The effective viscosity decays along with the energy decay when the stirring has stopped. The decay rate and decay timescale of the effective viscosity are experimentally determined, and empirical relations are given as functions of the kinematic viscosity and magnitude of the pulse forces. An overshoot effect is identified for Taylor-Reynolds number above a critical range *R*_*λ*_(0) > 20–25; a faster decay rate is observed at higher *R*_*λ*_(0), as the domain size constrained flow is more active within the skin depth near the free droplet surface and accelerates the dissipation. The findings of this work present an improvement in the viscosity measurements of liquid melts, help with the interpretation of physical mechanism using the oscillating droplet technique, and provide critical parameters as the benchmarks for future microgravity experiments and the development of fluid flow models.

## Methods

### Oscillating droplet experiments

The experiments were conducted using the ISS-EML facility in microgravity. The ISS-EML coil consists of a single set of electromagnetic coils superposing a minimal quadrupole field for positioning and a much stronger dipole field for heating or pulse excitation of a conducting droplet levitated in the center of the coil. A constant positioning power is maintained at 5.21 V, and the heating power is set in the range from 5.49–8.44 V for heating and pulse excitations. The specification of the ISS-EML coil was represented in^7^; the procedure and more details for an ISS-EML experiment were described in^2^. The Lorentz forces and induced convective flow under different conditions can be calculated and predicted using the surrogate magnetohydrodynamic (MHD) model^19,21^. The background fluid flow induced from the positioner force field is usually laminar, where the corresponding Reynolds numbers are around 100–150.

For a typical experimental cycle, a spherical sample of 6.0–6.5 mm in diameter was levitated, melted, and superheated when the heating power was on and then freely cooled by switching off the heater. The inert atmosphere is 450 mbar argon or helium (99.9999% purity), and the free cooling rate of the liquid droplet is approximately 20 K per second^3^. During the free cooling process, pulse excitations were applied by switching on the heating power for 0.1 s at multiple voltages to deform the droplet and initiate the oscillations over different temperature ranges. The temperature of the droplet was monitored by a pyrometer, and the motion of the droplet was captured by a top-view camera installed along the polar axis, and a side-view camera installed perpendicular to the equatorial axis.

**Fig. 9:**
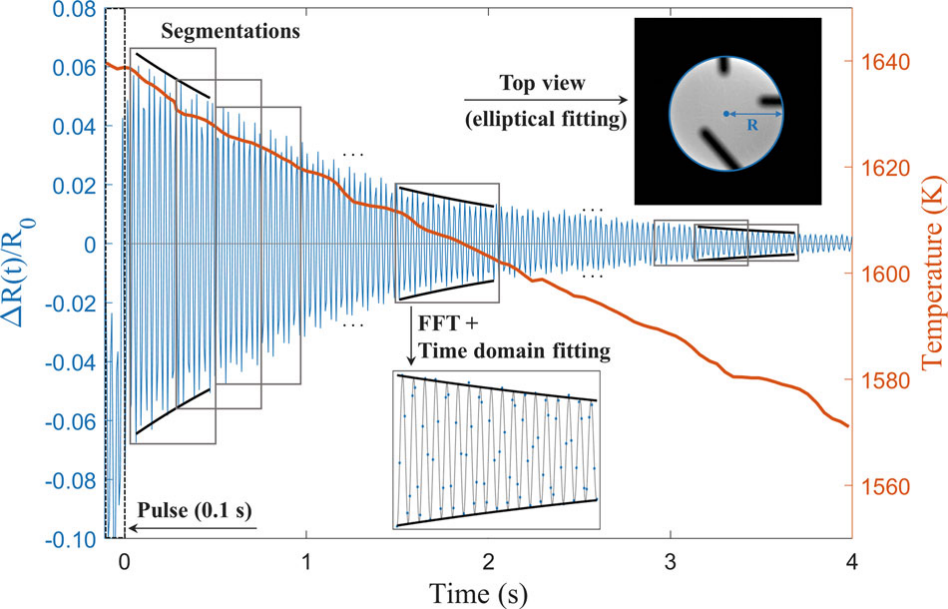
Typical damping curve of an oscillating droplet. The viscosities were measured at different time segments representing a temperature range between 5 and 10 K.

Define *t* = 0 as the origin of elapsed time after the pulse release. The top-view projection area of the droplet was subjected to an elliptical fitting to quantify the mean radius change Δ*R*(*t*) over both semi-axes as a function of time,

6 $${{\Delta }}R(t)/{R}_{0}\propto \cos (\omega t)\exp (-t/\tau )\,,$$

where *R*_0_ is the undeformed radius, *ω* is the angular frequency of the oscillation, and the parameter *τ* is the damping constant as a metric for the damping rate. The viscosity *μ* can be determined using Lamb’s equation,

7 $$\mu =\frac{\rho {{R}_{0}}^{2}}{(l-1)(2l+1)\tau }\,,$$

where oscillation mode *l* = 2 for an ISS-EML processed droplet, *ρ* is the temperature-dependent density of the molten alloy, and the damping constant *τ* is obtained by data fitting using Eq. (6). As shown in Fig. 9, the damping signal was segmented for each temperature range around 5–10 K ( ± 2.5% uncertainty around a constant viscosity) corresponding to a correlated time range around 0.3–0.6 s (45–90 frames/data points; 0.5–3% fitting error), then the fast Fourier transformation (FFT) and a time-domain fitting was applied to each segment to obtain the oscillation frequency *ω* and damping constant *τ*. Thus, the viscosity *μ* can be obtained using Eq. (7) in each segment as a function of temperature that changed with time. Using the segmenting setup described above, both the fitting error and uncertainty in temperature are optimized for the evaluation of the experimental data.

### Reporting summary

Further information on research design is available in the Nature Research Reporting Summary linked to this article.

## Supplementary information

Reporting Summary

## Data Availability

The data that support the findings of this study are available from the corresponding author upon request.
